# Genetic prioritisation of candidate drug targets for glaucoma through multi-trait and multi-omics integration

**DOI:** 10.1186/s40662-025-00442-4

**Published:** 2025-07-10

**Authors:** Jianqi Chen, Yangjiani Li, Yingting Zhu, Zhidong Li, Shitong Huang, Wenzhi Huang, Yuyao Ling, Jingying Liang, Yunxia Leng, Yehong Zhuo

**Affiliations:** 1 State Key Laboratory of Ophthalmology, Zhongshan Ophthalmic Center, Guangdong Provincial Key Laboratory of Ophthalmology and Visual Science, Sun Yat-Sen University, Guangzhou, 510060 Guangdong Province China; 2 Department of Ophthalmology, Guangzhou First People’s Hospital, Guangzhou Medical University, Guangzhou, 510180 Guangdong Province China

**Keywords:** Druggable targets, Glaucoma, Multi-omics, Multi-traits

## Abstract

**Background:**

Glaucoma causes permanent blindness. Current treatments have limited effectiveness, necessitating novel therapeutic strategies. We aimed to identify potential drug targets for glaucoma by integrating multi-trait and multi-omic analyses.

**Methods:**

We sourced druggable gene expression and protein abundance summary-level data from quantitative trait loci studies, and genetic associations with glaucoma from a large-scale multi-trait analysis. We employed proteome and transcriptome Mendelian randomization (MR) and colocalisation to identify potential therapeutic targets, glaucoma endophenotype MR to explore the potential mechanisms of identified associations, and phenome-wide MR to investigate possible adverse effects of candidate targets.

**Results:**

We identified *CPXM1* and *FLT4* as tier 1; *INSR* as tier 2; and *CPZ* and *PXDN* as tier 3 druggable genes. Genetically predicted higher levels of CPXM1 [odds ratio (OR): 0.86, 95% confidence interval (CI): 0.81–0.91, *P*_FDR_ < 0.001], FLT4 (OR: 0.74, 95% CI: 0.64 − 0.87, *P*_FDR_ = 0.033), INSR (OR: 0.58, 95% CI: 0.43 − 0.78, *P*_FDR_ = 0.042), and CPZ (OR: 0.55, 95% CI: 0.40 − 0.74, *P*_FDR_ = 0.033) were associated with decreased glaucoma risk while those of PXDN (OR: 1.33, 95% CI: 1.15 − 1.54, *P*_FDR_ = 0.033) with increased risk. The associations for CPXM1 (OR: 0.53, 95% CI: 0.39 − 0.73, *P* < 0.001) and FLT4 (OR: 0.86, 95% CI: 0.78 − 0.95, *P* = 0.005) were confirmed transcriptome-wide and colocalisation was confirmed for CPXM1 [posterior probability H4 (PPH_4_) = 0.940], FLT4 (PPH_4_ = 0.701), and INSR (PPH_4_ = 0.706). The protective effects of *CPXM1* and *CPZ* may be attributed to intraocular pressure-lowering activities. The risk associated with *PXDN* is due to its involvement in glaucomatous neuropathy. No significant adverse effects were identified.

**Conclusions:**

This study provides novel insights into glaucoma pathophysiology and promotes pharmaceutical target innovation.

**Supplementary Information:**

The online version contains supplementary material available at 10.1186/s40662-025-00442-4.

## Background

Glaucoma is a multifactorial eye disease characterised by the loss of retinal ganglion cells (RGCs) and axons, which results in irreversible optic nerve damage. It remains a prominent cause of permanent blindness and visual impairment worldwide [1], with a projected increase in its global incidence from 76 million in 2020 to 111.8 million in 2040 due to population ageing, representing a 47% increase [1–3].

The primary goal of glaucoma treatment is to protect the optic nerve. However, limited neuroprotective drugs are available to protect against glaucoma-related optic nerve damage [4]. Currently, intraocular pressure (IOP) is the sole factor that can be changed to reduce the risk of glaucoma progression [5]. Although various ophthalmic drugs have been approved for reducing IOP, they cannot fully regulate IOP, resulting in IOP fluctuations, and sometimes fail to achieve the desired IOP reduction, possibly due to differences in individual sensitivity [6]. Many IOP-lowering drugs are also associated with significant side effects, leading to poor patient compliance [7]. Moreover, existing therapy approaches have limited effectiveness in halting disease advancement [8]. Emerging treatments, such as stem cell and gene therapy, are still in the basic research stages [9]. Comprehensive research into the molecular networks associated with glaucoma may offer alternative treatments to enhance glaucoma therapy.

The primary flow of information in biological systems is from DNA (genome) to RNA (transcriptome) to proteins (proteome) [10]. Although genome-wide association studies (GWAS) have identified numerous genetic loci associated with glaucoma [11], these are located upstream of biological information and are far from viable therapeutic targets. Integrating large-scale genomic, transcriptomic, and proteomic data for glaucoma can aid in identifying novel biomarkers and determining the involvement of specific biomarkers in causal pathways. Methodical research using well-designed analytical frameworks into multi-omic biomarkers associated with glaucoma can also expedite the discovery of potential treatment targets [12]. The emergence of aptamer- and immunoassay-based platforms, such as the SomaScan platform, has enabled analysis of a wide range of proteins in large-scale GWAS datasets [13]. Protein biomarkers can also be precisely linked to coding genes in current GWAS datasets for transcriptomes [14]. By applying Mendelian randomization (MR)—a technique that utilises genetic variants as instrumental variables (IVs) to infer causal connections between exposures and outcomes—using quantitative trait loci (QTL) as IVs can offer supportive evidence for causal discoveries in drug target innovation [10].

In this study, by integrating large-scale GWAS data for protein QTLs (pQTLs), gene expression QTLs (eQTLs), and glaucoma multi-traits, we aimed to identify potential therapeutic targets for glaucoma.

## Methods

### Study design

**Fig. 1:**
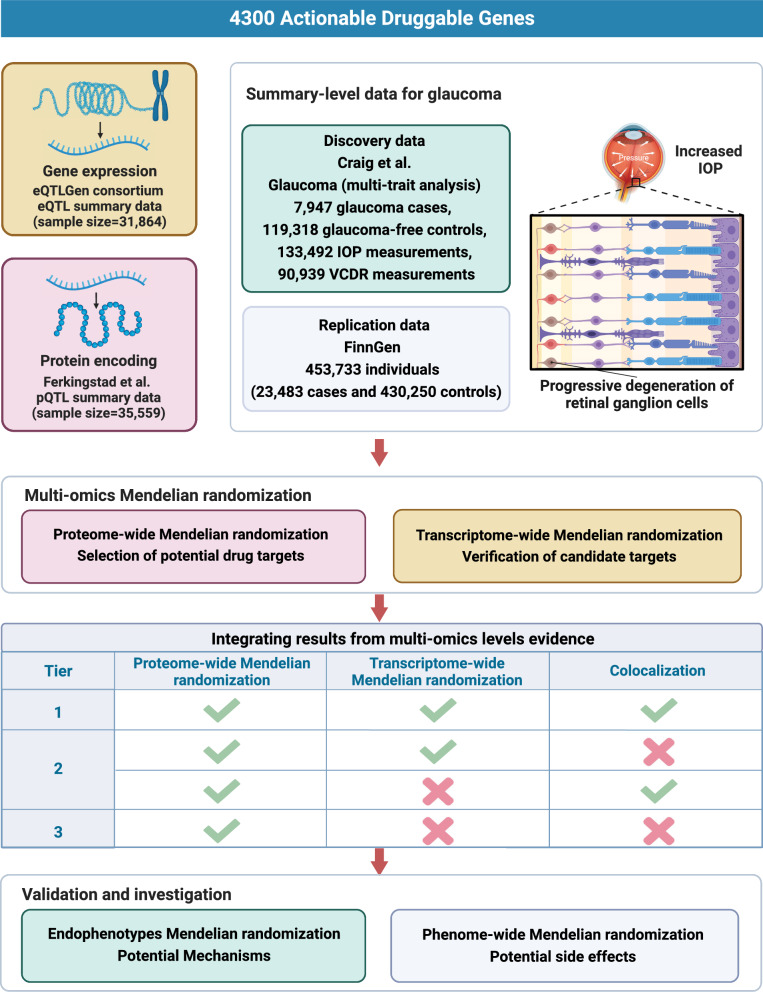
Study design. eQTL, expression quantitative trait loci; pQTL, protein quantitative trait loci; IOP, intraocular pressure; VCDR, vertical cup-to-disc ratio

We established a thorough analysis pipeline, as follows (Fig. 1): (1) to identify potential protein targets for glaucoma, we employed two-sample MR to analyse druggable proteomic GWAS data; (2) to validate the expression of the potential plasma protein-coding genes and confirm consistent associations, we analysed transcriptomic GWAS data using summary-data-based MR (SMR); (3) to validate the presence of shared causal loci between the identified proteins and glaucoma, we conducted colocalisation analysis; (4) to assess the strength of the obtained evidence, we classified potential drug targets based on the evidence from the above analyses; (5) to explore therapeutic effects underlying the identified targets, we employed glaucoma endophenotype MR validation; and (6) to investigate potential co-therapeutic and side effects of the identified targets, we conducted an MR-phenome-wide association study (MR-PheWAS).

### Identification of actionable druggable genes

The druggable genome was determined based on the parameters specified by Finan et al. and Li et al. [15, 16]. The dataset consists of 4,479 genes, categorised into three tiers based on their druggability. Tier 1 includes genes that encode targets of drugs either approved or undergoing clinical trials. Tier 2 encompasses genes that encode targets with notable sequence similarity to tier 1 proteins or those targeted by small drug-like molecules. Tier 3 comprises genes encoding secreted or extracellular proteins, genes from primary gene families that are druggable, and genes encoding proteins with partial similarity to tier 1 targets. After eliminating duplicates and all non-autosomal genes, 4,300 druggable genes were retained for further drug target analysis (Additional File 1).

### Data sources for QTLs

Data on pQTLs linked to plasma proteins were sourced from the SomaScan platform [13]. A total of 4,907 aptamers were identified from two initiatives involving 35,559 Icelanders [13]. The protein levels were adjusted for age, sex, and sample age using rank-inverse normalisation for each protein tested [13]. Gene expression data were obtained from the eQTLGen consortium, which offered a substantial sample size (n = 31,864) and enabled the identification of single-nucleotide polymorphisms (SNPs) associated with the expression of candidate genes [14]. The study focused on cis-eQTLs and cis-pQTLs to ensure the relevance of the genetic variants to changes in gene expression and protein encoding.

### Data sources for glaucoma and glaucoma endophenotypes

The summary statistics for glaucoma in Europeans were acquired from the study of Craig et al. [17], who employed a multi-trait approach to analyse data on glaucoma, IOP, and the vertical cup-to-disc ratio from the UK Biobank and the International Glaucoma Genetics Consortium. The GWAS results were derived from a sample of 7,947 glaucoma patients, 119,318 controls without glaucoma, 133,492 IOP measurements and 90,939 vertical cup-to-disc ratio measurements. This multi-trait method produces log odds ratio [log(OR)] estimates specific to glaucoma while minimising standard errors. This is similar to utilising genetically associated traits to conduct a typical glaucoma GWAS with a sample size corresponding to 20,582 glaucoma cases and 119,318 controls [18]. The FinnGen project dataset, including 23,483 cases and 430,250 controls, was used for replication to confirm and validate the findings [19]. Referencing a prior study [20], IOP (29,578 participants) and retinal nerve fibre layer (RNFL) thickness (31,434 participants) were selected as glaucoma endophenotypes to investigate the potential effects of candidate targets on IOP regulation and nerve protection [21, 22].

### Proteome‑wide MR analysis

To identify potential protein targets for glaucoma treatment, we conducted two-sample MR in the extensive plasma proteomic dataset. For the primary analysis, only cis-pQTLs were used as IVs for each protein. The outcome was glaucoma status from the multi-trait analysis in individuals of European ancestry. The Wald ratio method was applied to calculate ORs and their respective confidence intervals (CIs) for proteins with a single cis-pQTL, while the inverse-variance weighted method was used for proteins associated with multiple cis-pQTLs [23, 24].

The MR approach must adhere to these three assumptions: (1) the IV is correlated with the exposure, (2) the IV only influences the outcome through the exposure, and (3) the IV is not correlated with any confounding variables. Various measures were implemented to meet these assumptions [25]. First, we identified SNPs that displayed significant associations with the targeted exposure. Significant cis-pQTLs were identified by evaluating a genomic region centered around the relevant gene, with a range of ± 1,000 kb, using a threshold of *P* < 5.0 × 10^−8^. Subsequently, we conducted linkage disequilibrium (LD) clumping for these SNPs using PLINK (v.1.9). The clumping parameters were determined based on a default R^2^ threshold of 0.001 within a 10,000-kb frame. This stage involved estimating LD for European ancestries using data from the 1000 Genome Project. In cases where LD regions overlapped, only the most significant SNP was retained. Next, we evaluated the presence of horizontal pleiotropy through MR-Egger regression intercept analysis [26]. To account for potential reverse causation and ensure the correct direction of causality, we applied the Steiger filtering approach prior to MR analyses. This method tests whether each SNP explains more variance in the outcome than in the exposure. SNPs explaining more variance in the outcome than in the exposure were excluded from further analyses, as their associations may indicate reverse causation or invalid instruments. This filtering step strengthens the robustness of our causal inferences by reducing the risk of reverse causation bias [27]. Additionally, we eliminated SNPs directly linked to the outcome variable with a significance level of *P* < 5 × 10^–5^. The inclusion of cis-pQTLs in the analysis, together with subsequent colocalisation analyses, can contribute to mitigating genetic confounding caused by horizontal pleiotropy and LD, respectively [28]. The F-statistics of IVs were computed to eliminate those with values below 10, to ensure the robustness of the IVs [29].

The MR estimators quantified the impact of a one standard deviation increase in genetically predicted levels of plasma proteins on the risk for glaucoma. To account for multiple testing, *P* values < 0.05 corrected using the false discovery rate (FDR) method (*P*_FDR_) indicated statistical significance.

### Transcriptome‑wide MR analysis

To validate the plasma protein targets identified (*P*_FDR_ < 0.05), we used SMR to examine the association between the expression of candidate protein-coding genes in eQTLGen blood samples and glaucoma risk. Significant cis-eQTLs were identified by analysing a genomic region around the relevant gene, spanning ± 1000 kb, with a significance threshold of *P* < 5.0 × 10^−8^ (using default settings). SNPs that displayed allele frequency differences between the eQTL and glaucoma summary data and exceeded the 0.2 threshold were excluded [30]. Additionally, SNPs with minor allele frequencies ≤ 0.01 were removed.

The heterogeneity in the dependent instrument (HEIDI) test was subsequently employed to differentiate pleiotropy from linkage. We used the default parameters of *P* < 1.57 × 10^−3^ for the HEIDI testing, which is equivalent to a Chi-squared value (df = 1) of 10. Cases with *P* < 0.01 were considered likely to result from pleiotropy.

The SMR and HEIDI tests were conducted using the SMR software [31]. The significance criterion for *P* values in the SMR analysis was 0.05. The primary findings are also presented as ORs for disease per one standard deviation change in gene expression.

### Colocalisation analysis

Colocalisation analysis was conducted to assess whether the identified association between the candidate protein and glaucoma shared a common causal variant. Colocalisation analysis utilises a Bayesian model that evaluates five hypotheses with corresponding posterior probabilities: (1) no association with either trait (H_0_), (2) association with trait 1 only (H_1_), (3) association with trait 2 only (H_2_), (4) distinct causal variants for each trait (H_3_), and (5) a shared causal variant for both traits (H_4_) [32]. We used the default parameters, with a prior probability of 1 × 10^−4^ for an SNP associated with trait 1, 1 × 10^−4^ for association with trait 2, and 1 × 10^−5^ for association with both traits. We considered the relationship between the potential target protein and glaucoma to be colocalisation at PPH_4_ > 0.7 [33].

### Classification of the evidence of drug targets

Referencing a previously reported framework [10], we categorised the evidence of drug targets into three tiers (Fig. 1). Given that proteins are the final outcomes of genes, demonstrating causality at the protein level is crucial. Hence, candidate target genes in all three tiers of our categorisation had to be causally linked to glaucoma at the protein level. Tier 1, or high-confidence targets, demonstrate robust support across multiple levels of evidence, including proteome-wide MR, transcriptome-wide MR, and colocalisation analysis, and have comprehensive and consistent evidence from large-scale proteomic and transcriptomic data indicating strong associations with glaucoma through multiple rigorous analytical methods. Tier 2, or moderate-confidence targets, meet two of the three high-confidence criteria, with significant associations in proteome-wide MR and either transcriptome-wide MR or colocalisation analysis, and have substantial but not comprehensive evidence, indicating a moderate level of confidence in their relevance as drug targets. Tier 3, or preliminary targets, show significant association only in the proteome-wide MR analysis, indicating preliminary evidence for an association with glaucoma that requires further validation. Specifically, if the evidence from transcriptome-wide MR differed in direction from that in proteome-wide MR, the drug target was removed due to potential dual effects. This categorisation framework facilitates the prioritisation of drug targets based on the strength and breadth of supporting evidence, allowing for focused efforts on the most promising candidates for therapeutic development.

### Glaucoma endophenotype MR analysis

To explore the possible mechanisms underlying the associations between glaucoma and candidate genes, we conducted MR validations using the corresponding proteins as exposures and glaucoma endophenotypes (IOP and RNFL) as the outcomes [20]. The criteria for selecting IVs and the analytical procedure were the same as in the proteome-wide MR analysis.

### MR-PheWAS

To investigate the possible co-therapeutic and side effects of the putative targets, we conducted MR-PheWAS using genes encoding proteins as exposures and disease (trait) data from the UK Biobank as outcomes. The UK Biobank conducted disease GWASs using the scalable and accurate implementation of a generalised mixed-model (SAIGE v.0.29) technique to address the issue of imbalanced case–control ratios [34]. To ensure sufficient statistical power, we selected 783 diseases (traits) that had more than 500 cases for the MR-PheWAS. Causal effects were considered statistically significant at *P*_FDR_ < 0.05.

## Results

### Actionable circulating proteome and glaucoma

**Fig. 2:**
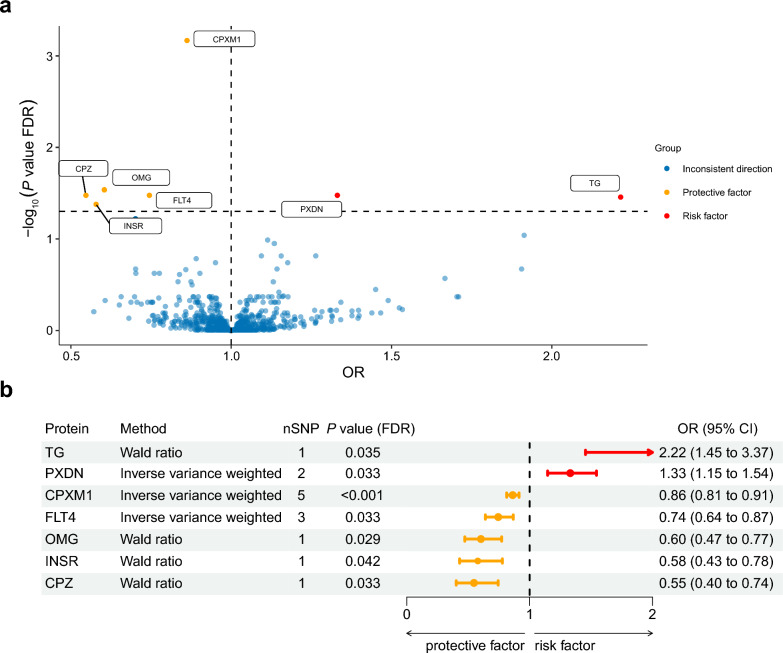
Proteome-wide Mendelian randomization for associations between druggable proteins and glaucoma in the discovery stage. **a** All druggable proteins evaluated. **b** Proteins remaining significant after FDR correction. FDR, false discovery rate; SNP, single-nucleotide polymorphism; OR, odds ratio; CI, confidence interval

In the discovery sample, 94 proteins were significantly associated with glaucoma. After FDR correction, associations remained significant for seven proteins (Fig. 2 and Additional File 2). Among them, TG (OR: 2.22, 95% CI: 1.45–3.37, *P*_FDR_ = 0.035) and PXDN (OR: 1.33, 95% CI: 1.15–1.54, *P*_FDR_ = 0.033) were associated with an increased risk of glaucoma, whereas CPXM1 (OR: 0.86, 95% CI: 0.81–0.91, *P*_FDR_ < 0.001), FLT4 (OR: 0.74, 95% CI: 0.64–0.87, *P*_FDR_ = 0.033), OMG (OR: 0.60, 95% CI: 0.47–0.77, *P*_FDR_ = 0.029), INSR (OR: 0.58, 95% CI: 0.43–0.78, *P*_FDR_ = 0.042), and CPZ (OR: 0.55, 95% CI: 0.40–0.74, *P*_FDR_ = 0.033) were associated with a decreased risk of glaucoma. In the replication study with the FinnGen dataset, most of the associations showed directional trends consistent with those found in the discovery phase, except for TG (Additional File 3).

### Candidate gene expression and glaucoma

**Fig. 3:**
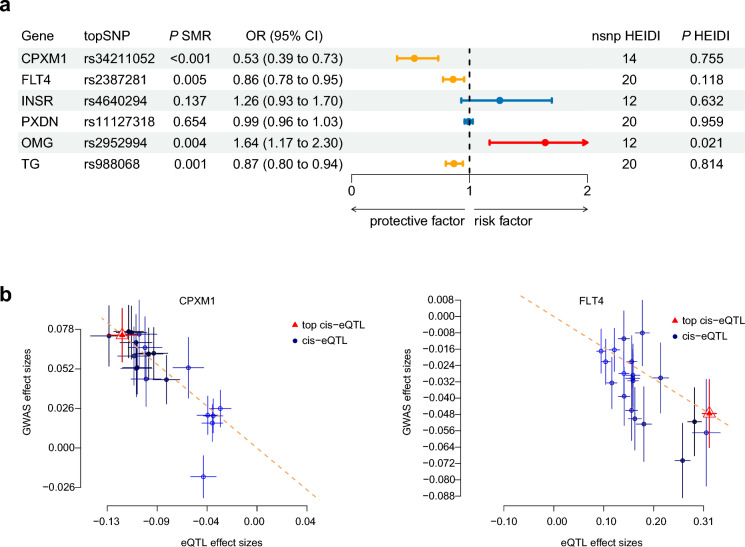
Transcriptome-wide Mendelian randomization for associations between candidate druggable genes and glaucoma in the discovery stage. **a** The forest plot shows ORs and 95% CIs of the gene-glaucoma association derived from the SMR test. **b** The scatter plots illustrate the relationship between eQTL effect sizes and GWAS effect sizes for SNPs in the cis region of representative genes, with the top cis-eQTL highlighted. SNP, single-nucleotide polymorphism; SMR, summary-data-based Mendelian randomization; OR, odds ratio; CI, confidence interval; HEIDI, heterogeneity in the dependent instrument; GWAS, genome-wide association study; eQTL, expression quantitative trait loci

**Fig. 4:**
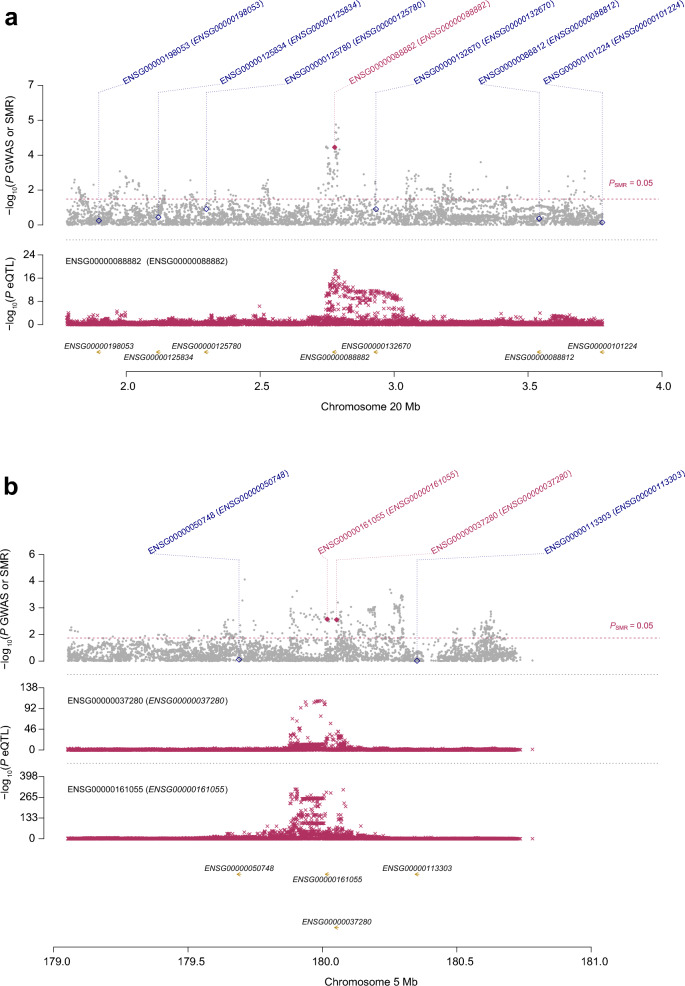
SMR for associations between (**a**) *CPXM1* and (**b**) *FLT4* and glaucoma in the discovery stage. SMR, summary-data-based Mendelian randomization; GWAS, genome-wide association study; eQTL, expression quantitative trait loci

We mapped the seven proteins to six coding genes in the eQTLGen consortium dataset. Among them, *CPXM1* (OR: 0.53, 95% CI: 0.39 − 0.73, *P* < 0.001) and *FLT4* (OR: 0.86, 95% CI: 0.78 − 0.95, *P* = 0.005) exhibited associations with glaucoma consistent with those of the corresponding proteins (Figs. 3 and 4). In contrast, *INSR* and *PXDN* gene expressions were not significantly associated with risk of glaucoma. The associations for *OMG* (OR: 1.64, 95% CI: 1.17 − 2.30, *P* = 0.004) and *TG* (OR: 0.87, 95% CI: 0.80 − 0.94, *P* = 0.001) demonstrated inconsistent directions with glaucoma risk relative to those of the corresponding proteins and were therefore removed from further analysis. In the replication sample, the results from the discovery sample were replicated for *CPXM1* (OR: 0.62, 95% CI: 0.47 − 0.83, *P* = 0.001; Additional File 4).

### Colocalisation of the putative target proteins with glaucoma

**Fig. 5:**
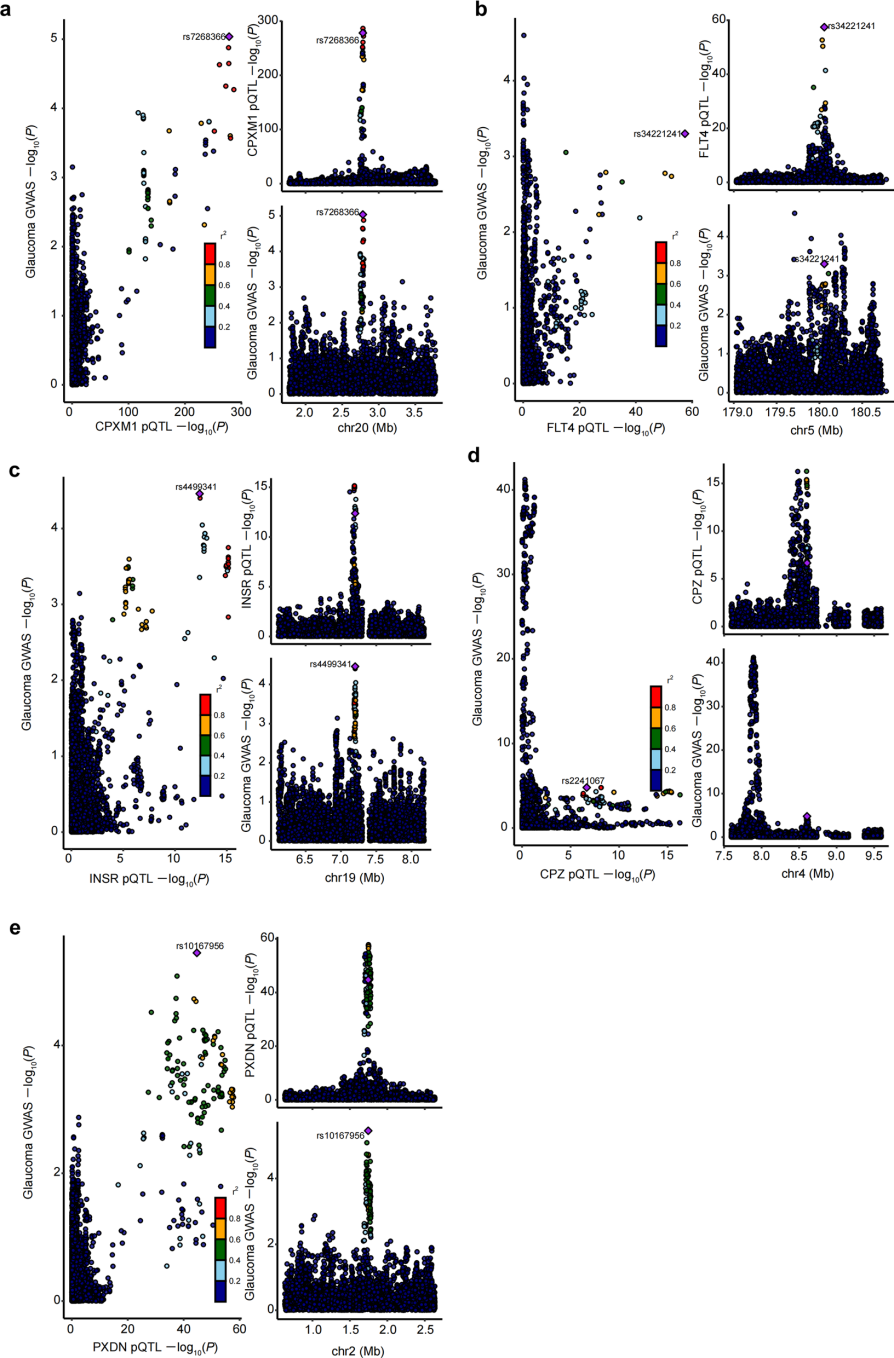
Colocalisation of the putative proteins with glaucoma. **a** CPXM1, **b** FLT4, **c** INSR, **d** CPZ, and **e** PXDN. GWAS, genome-wide association study; pQTL, protein quantitative trait loci

Among the five proteins, CPXM1 (PPH_4_ = 0.940), FLT4 (PPH_4_ = 0.701), and INSR (PPH_4_ = 0.706) were found to colocalise with glaucoma, while CPZ (PPH_4_ < 0.001) and PXDN (PPH_4_ = 0.447) did not show evidence of colocalisation with glaucoma (Fig. 5).

### Classification of the evidence

Tier 1 proteins included CPXM1 and FLT4, both of which passed MR, SMR, and colocalisation. Tier 2 proteins included only INSR, which passed MR and colocalisation but did not provide evidence from SMR. Tier 3 proteins included CPZ and PXDN, which passed MR but not the SMR and colocalisation analyses (PXDN: no evidence; CPZ: no available gene expression data).

### Glaucoma endophenotype MR results

In the validation using glaucoma endophenotype data, CPXM1 (β = − 0.20, 95% CI: − 0.37 to − 0.04, *P* = 0.015) and CPZ (β = − 1.90, 95% CI: − 2.84 to − 0.97, *P* < 0.001) were associated with decreased IOP, whereas PXDN was associated with decreased RNFL thickness (β = − 0.53, 95% CI: − 1.05 to − 0.02, *P* = 0.043) (Table 1).

**Table 1:** Mendelian randomization for associations between candidate protein targets and glaucoma endophenotypes

Glaucoma endophenotype	Candidate protein	Method	No. of SNPs	β (95% CI)	*P* value
IOP	CPXM1	Inverse-variance weighted	4	− 0.20 (− 0.37 to − 0.04)	**0.015**
	CPZ	Wald ratio	1	− 1.90 (− 2.84 to − 0.97)	** < 0.001**
	FLT4	Inverse-variance weighted	3	− 0.26 (− 1.21 to 0.68)	0.585
	INSR	Wald ratio	1	− 0.08 (− 0.82 to 0.66)	0.833
	PXDN	Inverse-variance weighted	2	− 0.10 (− 0.51 to 0.31)	0.632
RNFL thickness	CPXM1	Inverse-variance weighted	5	0.06 (− 0.16 to 0.27)	0.612
	CPZ	Wald ratio	1	0.78 (− 0.29 to 1.85)	0.152
	FLT4	Inverse-variance weighted	3	0.06 (− 0.54 to 0.65)	0.853
	INSR	Wald ratio	1	− 0.84 (− 1.87 to 0.18)	0.107
	PXDN	Inverse-variance weighted	2	− 0.53 (− 1.05 to − 0.02)	**0.043**

*CI* = confidence interval; *IOP* = intraocular pressure; *RNFL* = retinal nerve fibre layer; *SNP* = single-nucleotide polymorphism. *P* < 0.05 indicates statistical significance (bold font values)

### MR-PheWAS results

**Fig. 6:**
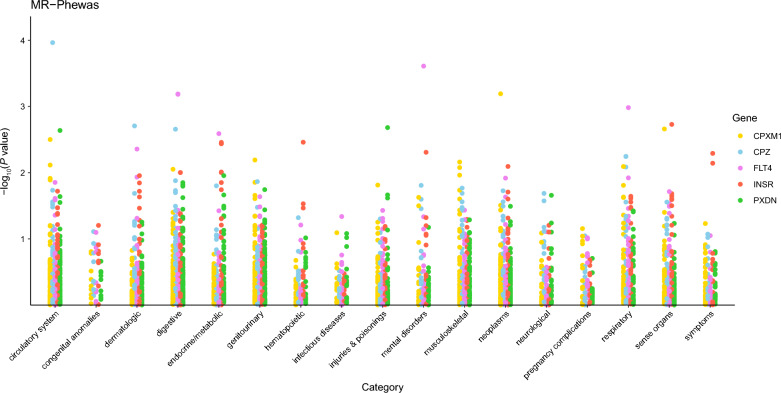
Mendelian randomization phenome-wide association study results. Manhattan plot of the associations between candidate targets and traits in UKB-SAIGE

Among 783 different traits across 17 categories from the UKB-SAIGE database, CPXM1 was linked to 34, FLT4 to 34, INSR to 46, CPZ to 44, and PXDN to 25 phenotypes (Fig. 6, Additional Files 5 to 14). These phenotypes may either act as co-therapeutic effects for the putative proteins or be related to adverse effects. However, none of the discovered associations between target plasma proteins and various phenotypes remained significant after FDR correction (Additional Files 5 to 14).

## Discussion

In this study, we conducted multi-omics analyses for multi-trait data to identify and prioritise therapeutic targets for glaucoma. This study revealed the following key findings. Among actionable druggable genes, *CPXM1* and *FLT4* exhibited protective roles in glaucoma, supported by tier 1 evidence. *INSR* was also associated with decreased risk for glaucoma, supported by tier 2 evidence. Additionally, *CPZ* and *PXDN* demonstrated protective and risk-associated roles in glaucoma, respectively, supported by tier 3 evidence. The protective roles of *CPXM1* and *CPZ* were associated with decreased IOP, whereas the risk associated with *PXDN* was potentially linked to glaucoma neuropathy.

Current investigational therapies for glaucoma predominantly focus on IOP-lowering mechanisms, including Rho-kinase inhibitors [35], nitric oxide (NO) donors [36], adenosine receptor modulators [37], small-interfering RNAs (siRNAs) [38], C-type natriuretic peptides (CNP) [39], and LIM domain kinase inhibitors [40]. In parallel, non-IOP lowering strategies aimed at neuroprotection are also in development, such as various neurotrophic factors and dietary supplements [41, 42]. Most of these targets have been proposed based on preclinical findings from animal models or in vitro systems. Therapeutic targets identified through animal experiments frequently face significant challenges during clinical translation, resulting in high attrition rates, which makes drug development a time-consuming and costly procedure. In contrast, our study leveraged MR analyses that integrate large-scale QTL data with GWAS for glaucoma. This approach enables the identification of target-disease associations with a degree of causal inference similar to randomised controlled trials [43], thereby enhancing the translational relevance of the prioritised targets. Importantly, many of the identified targets in our study have not yet been recognised in the current drug development pipelines for glaucoma, highlighting a set of novel and potentially actionable therapeutic targets.

*FLT4*, a candidate target gene with tier 1 evidence for association with glaucoma and for druggability, did not exhibit a significant association with IOP or RNFL. However, its protective effect against glaucoma might be attributed to neuroprotective activity. Specifically, the *FLT4* gene encodes the vascular endothelial growth factor receptor 3 (VEGF-R3) [44]. In humans, VEGF-R3 is bound and activated by vascular endothelial growth factor-D (VEGF-D) [45]. The retina exhibits a strong connection between RGCs and capillaries [46]. This interaction between vascular and neuronal components is possibly modulated by members of the VEGF family [47–50]. In a prior study, RGC injury led to decreased VEGF-D expression in neurons, accompanied by capillary damage [51]. VEGF-D supplementation enhanced the survival of RGCs, which was essential for preventing the development of collapsed and acellular capillaries [51]. VEGF-D generated by RGCs likely affects their autocrine receptors, as downregulation of VEGF-R3 expression selectively in RGCs inhibited the protective effect of VEGF-D [51]. This process could help protect RGCs from damage caused by N-methyl-D-aspartate and limit the creation of empty sleeves [51]. This finding may partially account for the protective role of *FLT4* in glaucoma and may also have significant therapeutic implications in effectively enhancing the integrity of neurons and blood vessels.

*CPXM1*, another candidate target gene with tier 1 evidence for association with glaucoma and tier 3A evidence for druggability, has not been extensively studied. CPXM1 is a member of the M14 family of carboxypeptidases, upregulated in the cells of Schlemm’s canal, a conduit that exhibits both vascular and lymphatic traits [52, 53]. The endothelial cells that make up the inner wall of Schlemm’s canal are jointly responsible for controlling the resistance to fluid outflow and regulating IOP [52]. The protective effect of *CPXM1* against glaucoma could be attributed to its IOP-lowering role according to our glaucoma endophenotypes validation. This protective effect of *CPXM1* may be associated with its involvement in the extracellular matrix remodelling process [53, 54].

*INSR* was identified as a tier 2 candidate gene for glaucoma with tier 1 evidence for druggability. Although its specific role in glaucoma was not identified in the glaucoma endophenotype analysis, its protective effect may be due to neuroprotective activity. INSR, an insulin receptor that is a crucial regulator of peripheral metabolism, significantly influences neuronal cell survival and the development, maintenance, and activity of synapses [55, 56]. INSR expression is present in several parts of the central nervous system, as well as in the retina [57]. At the individual neuron level, INSR controls synaptic function by regulating the trafficking of neurotransmitter receptors. It is also involved in maintaining synapses and the formation of dendritic arbors [56, 58]. Reduced INSR signalling has been linked to various neurological conditions, including glaucoma, and stimulating INSR signalling has been suggested as a possible therapeutic option [55, 59]. The protective role of INSR in glaucoma is likely mediated through pathways involved in RGC dysfunction. Insulin enhances RGC survival in response to various stressors that induce apoptosis, with PI3K and Akt playing crucial roles in facilitating insulin-induced neuronal survival [60]. Other potential pathways include mitochondrial biogenesis and function, regenerating retracted RGC dendrites and enhancing synaptic plasticity, reducing the levels of Tau phosphorylation, and targeting amyloid-β deposition [60].

*CPZ*, a tier 3 target gene for glaucoma with tier 3B evidence for druggability, also exhibited a protective role in glaucoma, associated with IOP reduction according to the glaucoma endophenotype analysis. CPZ is a metallocarboxypeptidase that potentially targets extracellular peptides or proteins containing basic residues at the C-terminus, modulating their interaction with other compounds. Increased expression of carboxypeptidase Z in living organisms could impact cell adhesion or movement, potentially playing a role in regulating IOP [61].

*PXDN*, a tier 3 target gene with tier 3A evidence for druggability, was the only putative target gene associated with increased glaucoma risk. Based on our findings in the glaucoma endophenotype analysis, its negative effect may be associated with its role in glaucoma neuropathy. PXDN encodes a haem peroxidase that participates in the production of extracellular matrix and may contribute to pathological fibrotic reactions [62]. An increase in fibrosis-related signalling may result in increased extracellular matrix remodelling, making the lamina cribrosa more vulnerable to injury caused by increased IOP due to greater tissue stiffness and crosslinking of the extracellular matrix [63]. Regulating *PXDN* may be beneficial in protecting against glaucoma-related neurological damage.

The main strength of our study is that we combined multi-trait and multi-omic evidence, which enhanced the causal association between actionable druggable genes and glaucoma. Moreover, the use of GWAS datasets with substantial sample sizes significantly enhanced the statistical power of our analyses. Nonetheless, our study also has some limitations. First, we could not fully explore all druggable genes for glaucoma due to the limited availability of pQTLs and specific genes in the eQTL dataset, such as *CPZ*, which restricted our ability to prioritise gene tiers. The differentiation between glaucoma subtypes was not possible due to the limitations inherent in the summary-level GWAS data, which did not provide subtype-specific association results. This limitation stems from the original GWAS, where only a small proportion of glaucoma cases had documented subtypes in the data source utilised by Craig et al. [17]. Nevertheless, it is well-established that primary open-angle glaucoma (POAG) constitutes the majority of glaucoma cases within the dataset population, suggesting that our findings predominantly reflect the biology of POAG [64]. Second, although we applied MR to infer potential causal relationships between candidate targets and glaucoma risk, we acknowledge that MR cannot eliminate the possibility of reverse causation, particularly in the context of complex diseases. To mitigate this, we performed Steiger filtering to ensure that the selected genetic instruments explained more variance in the exposure than in the outcome, thereby reducing the likelihood of reverse causation bias. Nonetheless, the identified associations should be interpreted cautiously, as some candidate targets might still reflect downstream consequences or compensatory responses to disease development rather than true causal drivers. Further functional validation will be necessary to confirm the therapeutic relevance of these candidates. To fully establish the therapeutic potential of candidate targets, potential follow-up experiments include CRISPR/Cas9 knockout or overexpression studies in relevant ocular cell types (e.g., trabecular meshwork cells, RGCs) and animal models of glaucoma to observe phenotypic effects. These approaches would be instrumental in elucidating the mechanisms through which these targets influence glaucoma and assessing their suitability as therapeutic targets. Third, we used two complementary tiering frameworks: one for assessing the druggability of genes (based on pharmacological criteria) and another for prioritising candidate targets based on the current MR framework. We recognise that drug target classification is inherently multidimensional, and a single target may occupy different tiers under each framework. Therefore, therapeutic prioritisation should be considered in an integrated manner. A further limitation is that sex-specific effects could not be assessed due to the use of GWAS summary data without access to individual-level information. In addition, our analyses were restricted to individuals of European ancestry to minimise population stratification; as a result, the generalisability of our findings to other ancestral groups may be limited.

## Conclusion

By using a comprehensive analysis pipeline, we identified five potential therapeutic targets for glaucoma: two tier 1 genes (*CPXM1* and *FLT4*), one tier 2 gene (*INSR*), and two tier 3 genes (*CPZ* and *PXDN*). These findings provide novel insights into the pathological mechanisms underlying glaucoma and promote pharmaceutical target innovation.

## Supplementary Information

Additional file 1. Table S1. The list of 4300 druggable genes included in the study.

Additional file 2. Table S2. The proteome-wide Mendelian randomization analysis in the discovery sample.

Additional file 3. Figure S1. Validation of candidate protein targets in the replication sample. SNP, single-nucleotide polymorphism; OR, odds ratio; CI, confidence interval.

Additional file 4. Figure S2. Transcriptome-wide Mendelian randomization for associations between candidate druggable genes and glaucoma in the replication sample. a All candidate genes. b The significant gene. SNP, single-nucleotide polymorphism; SMR, summary-data-based Mendelian randomization; OR, odds ratio; CI, confidence interval; HEIDI, heterogeneity in the dependent instrument; GWAS, genome-wide association study; eQTL, expression quantitative trait loci.

Additional file 5. Figure S3. Manhattan plot of the associations of CPXM1 with traits in UKB-SAIGE in the Mendelian randomization phenome-wide association study.

Additional file 6. Figure S4. Manhattan plot of the associations of FLT4 with traits in UKB-SAIGE in the Mendelian randomization phenome-wide association study.

Additional file 7. Figure S5. Manhattan plot of the associations of INSR with traits in UKB-SAIGE in the Mendelian randomization phenome-wide association study.

Additional file 8. Figure S6. Manhattan plot of the associations of CPZ with traits in UKB-SAIGE in the Mendelian randomization phenome-wide association study.

Additional file 9. Figure S7. Manhattan plot of the associations of PXDN with traits in UKB-SAIGE in the Mendelian randomization phenome-wide association study.

Additional file 10. Table S3. The associations of CPXM1 with traits in UKB-SAIGE in the Mendelian randomization phenome-wide association study.

Additional file 11. Table S4. The associations of FLT4 with traits in UKB-SAIGE in the Mendelian randomization phenome-wide association study.

Additional file 12. Table S5. The associations of INSR with traits in UKB-SAIGE in the Mendelian randomization phenome-wide association study.

Additional file 13. Table S6. The associations of CPZ with traits in UKB-SAIGE in the Mendelian randomization phenome-wide association study.

Additional file 14. Table S7. The associations of PXDN with traits in UKB-SAIGE in the Mendelian randomization phenome-wide association study.

## Data Availability

The datasets used and analysed in this study are available from the corresponding author on reasonable request.

## Abbreviations

RGC: Retinal ganglion cell
IOP: Intraocular pressure
GWAS: Genome-wide association study
MR: Mendelian randomization
IV: Instrumental variable
QTL: Quantitative trait loci
pQTL: Protein quantitative trait loci
eQTL: Expression quantitative trait loci
SMR: Summary-data-based Mendelian randomization
MR-PheWAS: Mendelian randomization-phenome-wide association study
SNP: Single-nucleotide polymorphism
OR: Odds ratio
RNFL: Retinal nerve fibre layer
CI: Confidence interval
LD: Linkage disequilibrium
FDR: False discovery rate
HEIDI: Heterogeneity in the dependent instrument
VEGF: Vascular endothelial growth factor
