# Supplementary figures and images for: Genetic prioritisation of candidate drug targets for glaucoma through multi-trait and multi-omics integration
Source: Eye Vis (Lond). 2025 Jul 10;12:26. doi: 10.1186/s40662-025-00442-4 (PMC12243406; doi:10.1186/s40662-025-00442-4)

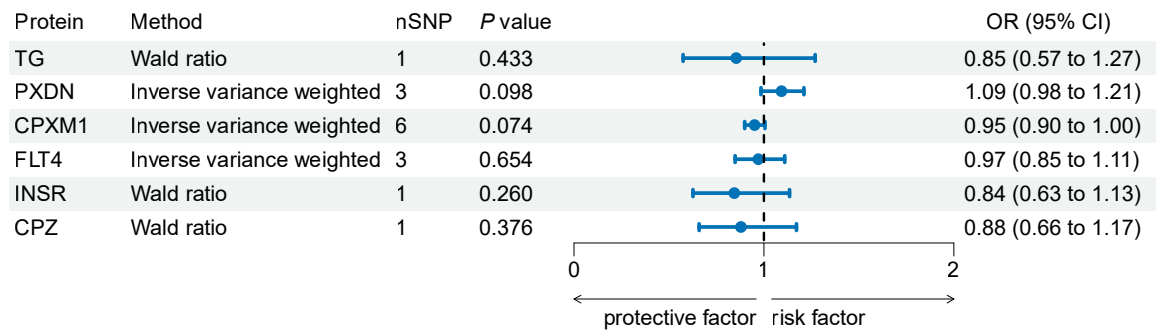

Supplement: Supplementary file 3 — Additional file 3. Figure S1. Validation of candidate protein targets in the replication sample. SNP, single-nucleotide polymorphism; OR, odds ratio; CI, confidence interval. [file 40662_2025_442_MOESM3_ESM.pdf]

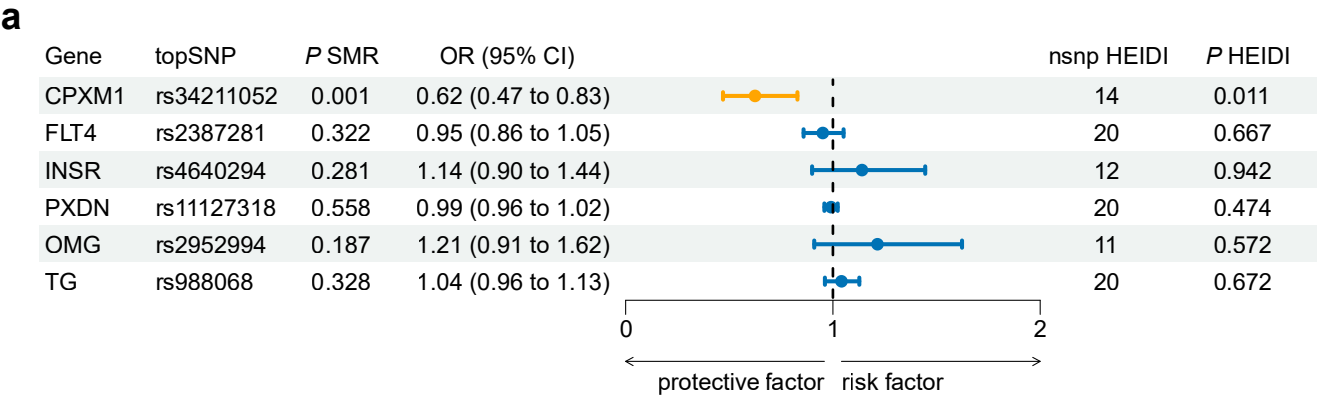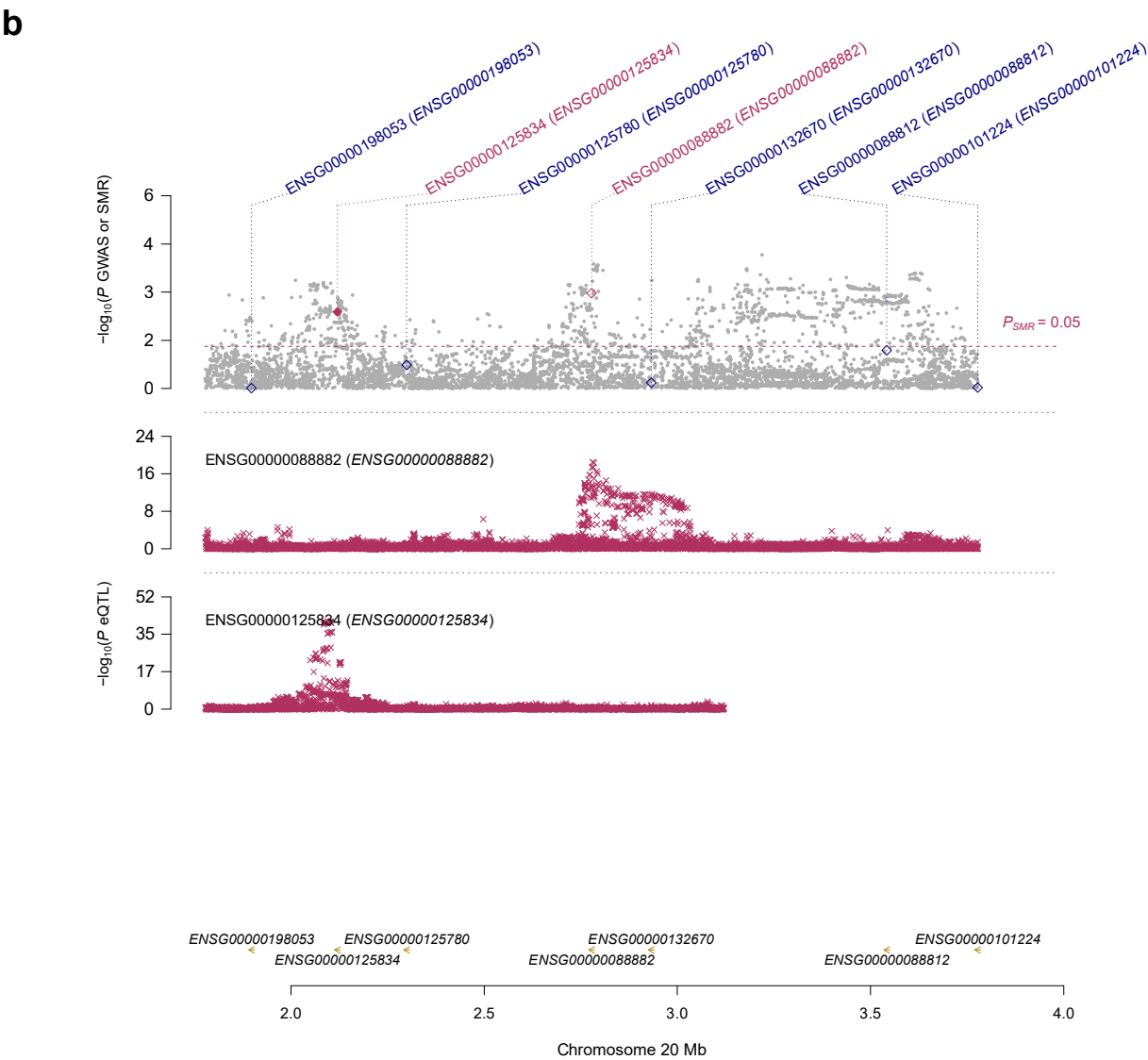

Supplement: Supplementary file 4 — Additional file 4. Figure S2. Transcriptome-wide Mendelian randomization for associations between candidate druggable genes and glaucoma in the replication sample. a All candidate genes. b The significant gene. SNP, single-nucleotide polymorphism; SMR, summary-data-based Mendelian randomization; OR, odds ratio; CI, confidence interval; HEIDI, heterogeneity in the dependent instrument; GWAS, genome-wide association study; eQTL, expression quantitative trait loci. [file 40662_2025_442_MOESM4_ESM.pdf]

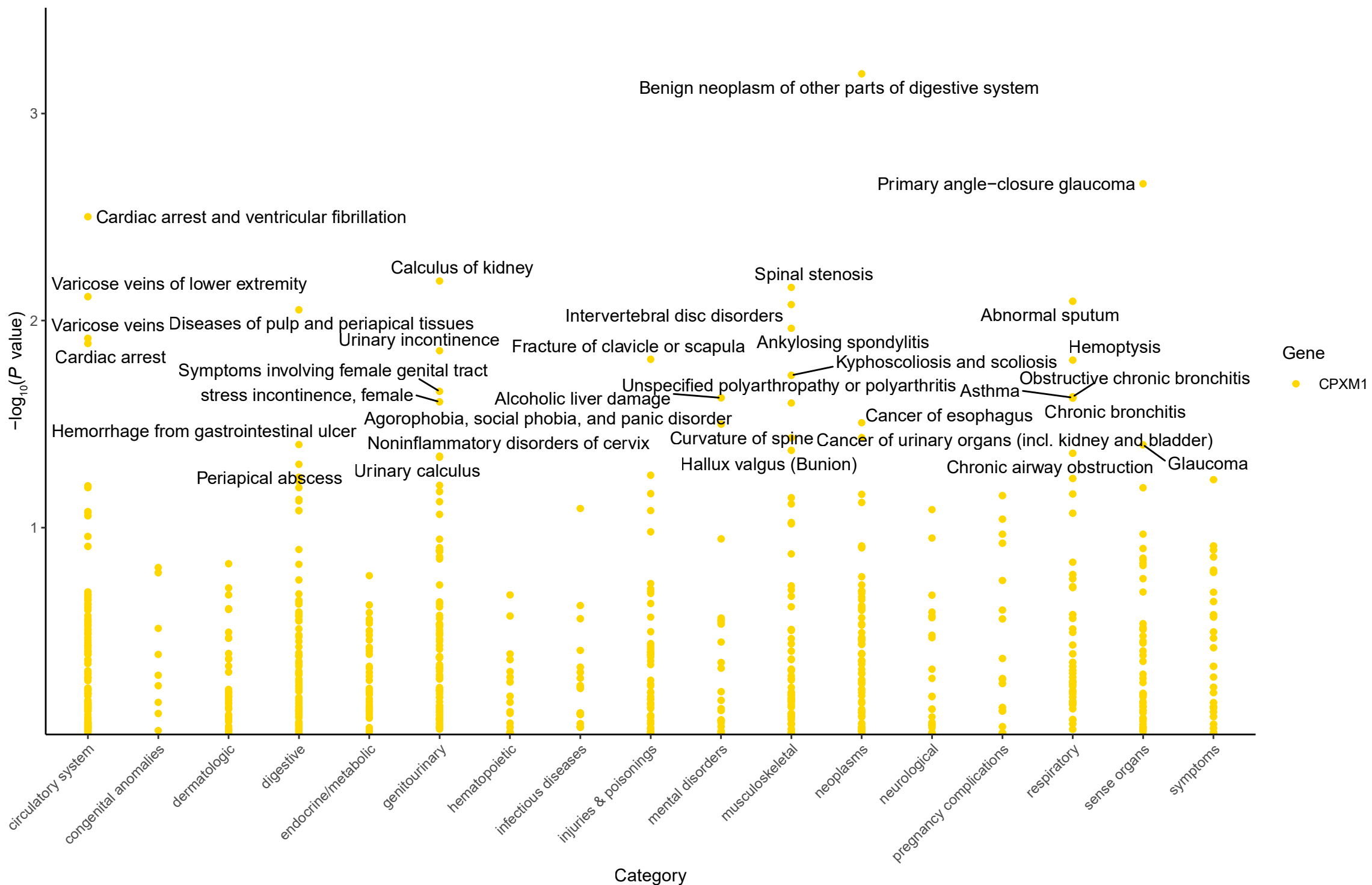

Supplement: Supplementary file 5 — Additional file 5. Figure S3. Manhattan plot of the associations of CPXM1 with traits in UKB-SAIGE in the Mendelian randomization phenome-wide association study. [file 40662_2025_442_MOESM5_ESM.pdf]

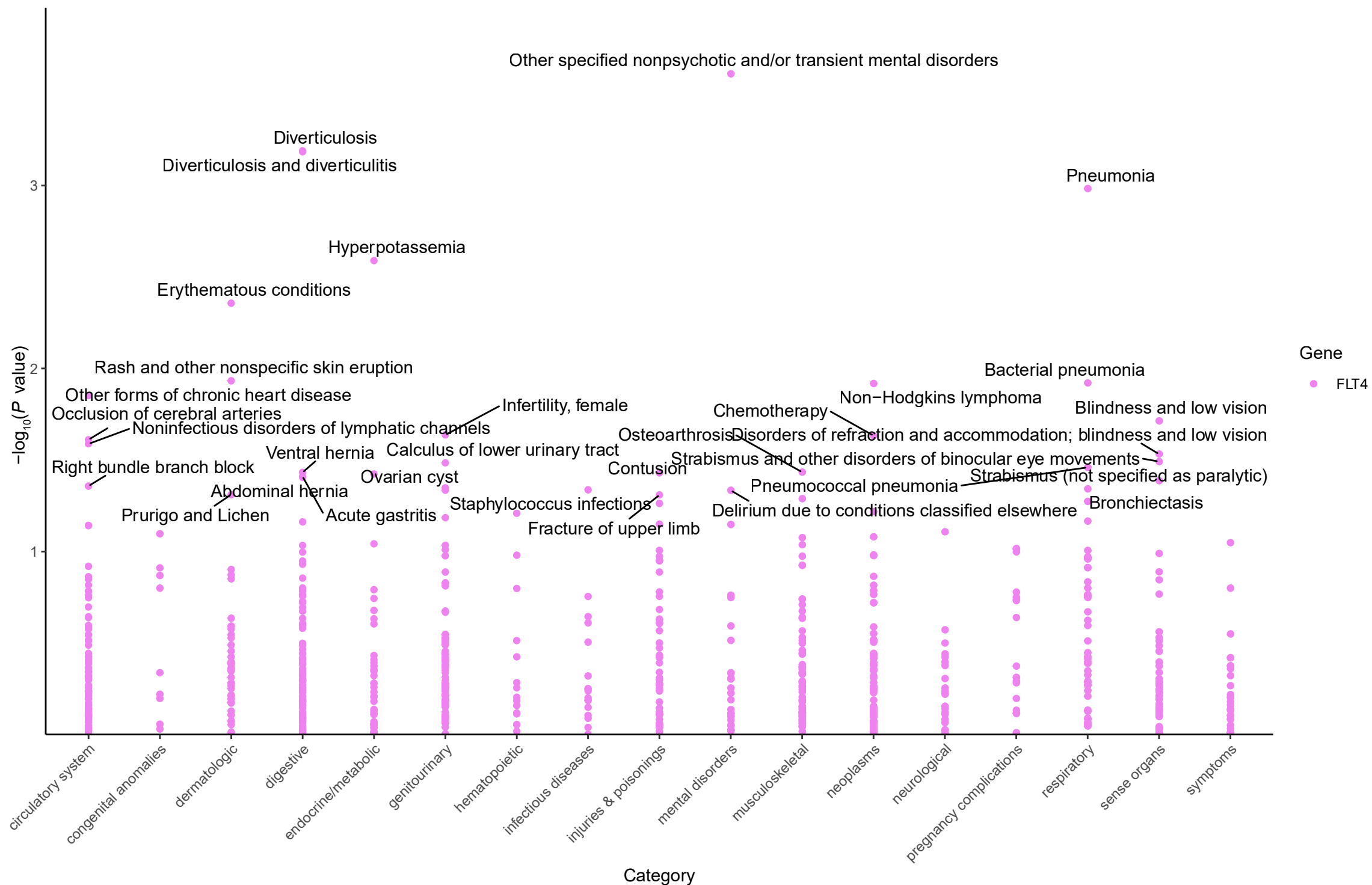

Supplement: Supplementary file 6 — Additional file 6. Figure S4. Manhattan plot of the associations of FLT4 with traits in UKB-SAIGE in the Mendelian randomization phenome-wide association study. [file 40662_2025_442_MOESM6_ESM.pdf]

## INSR MR-Phewas

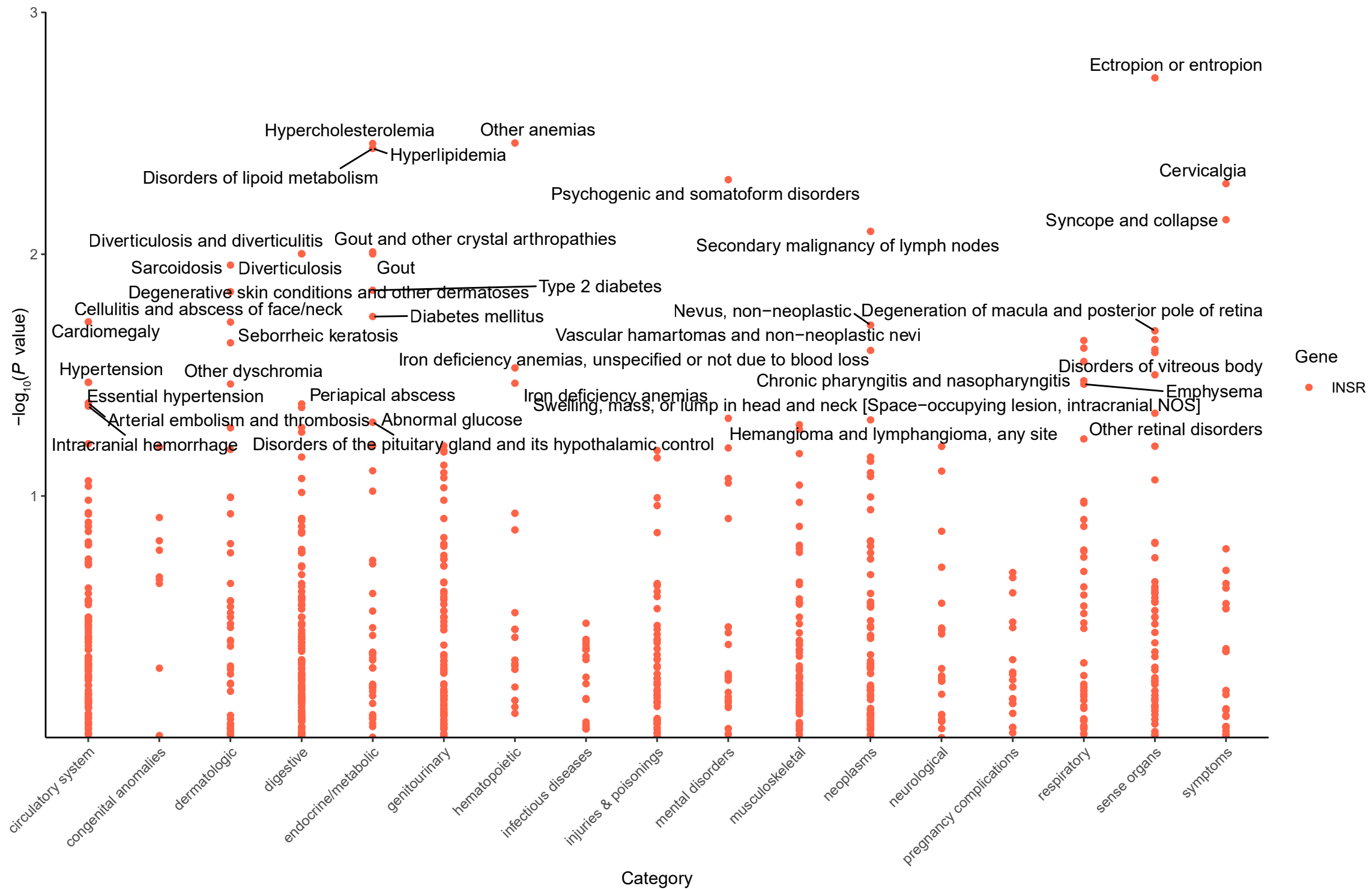

Supplement: Supplementary file 7 — Additional file 7. Figure S5. Manhattan plot of the associations of INSR with traits in UKB-SAIGE in the Mendelian randomization phenome-wide association study. [file 40662_2025_442_MOESM7_ESM.pdf]

# CPZ MR-Phewas

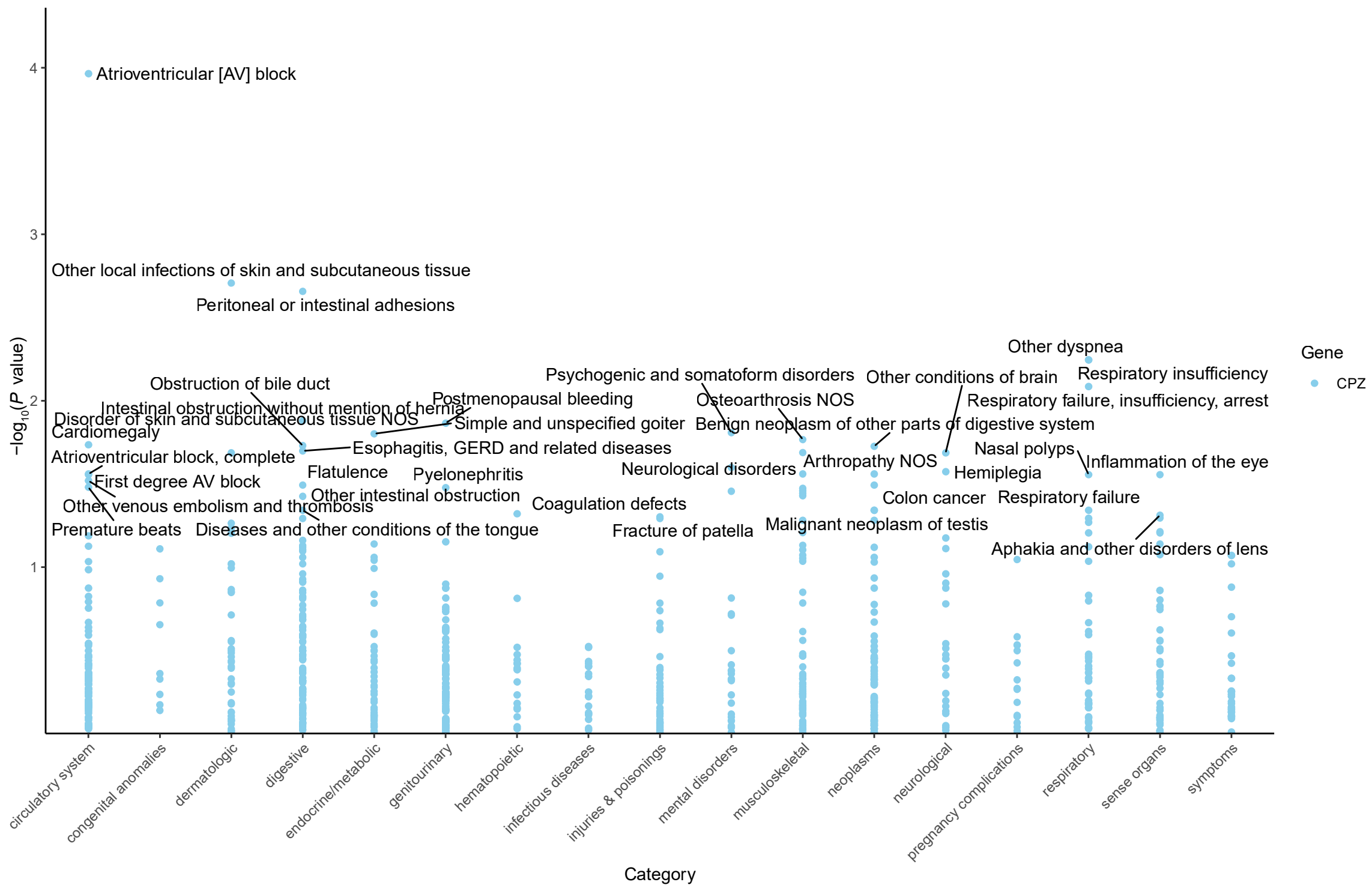

Supplement: Supplementary file 8 — Additional file 8. Figure S6. Manhattan plot of the associations of CPZ with traits in UKB-SAIGE in the Mendelian randomization phenome-wide association study. [file 40662_2025_442_MOESM8_ESM.pdf]

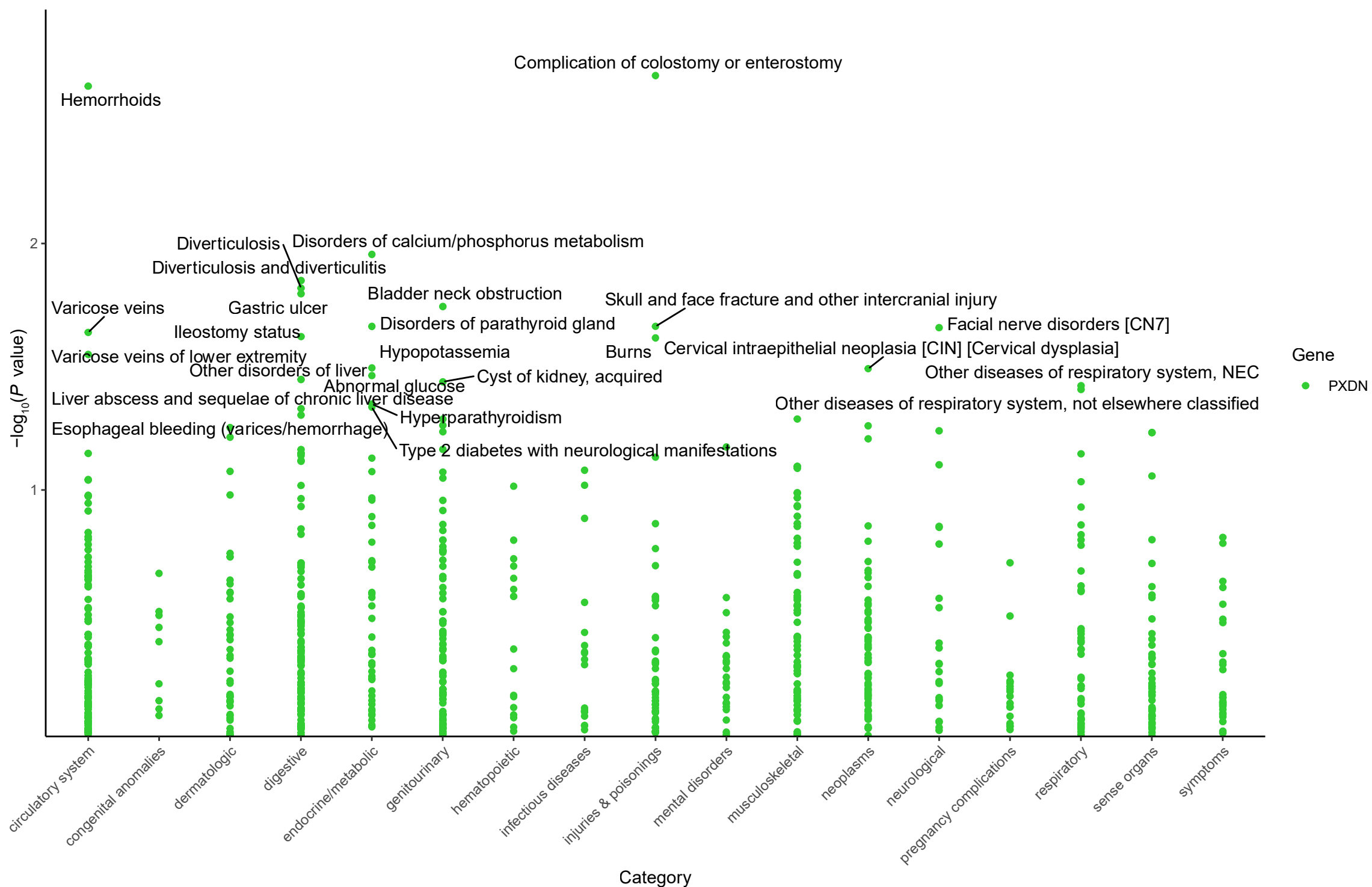

Supplement: Supplementary file 9 — Additional file 9. Figure S7. Manhattan plot of the associations of PXDN with traits in UKB-SAIGE in the Mendelian randomization phenome-wide association study. [file 40662_2025_442_MOESM9_ESM.pdf]
